# Fast Registration Algorithm for Laser Point Cloud Based on 3D-SIFT Features

**DOI:** 10.3390/s25030628

**Published:** 2025-01-22

**Authors:** Lihong Yang, Shunqin Xu, Zhiqiang Yang, Jia He, Lei Gong, Wanjun Wang, Yao Li, Liguo Wang, Zhili Chen

**Affiliations:** 1 School of Photoelectric Engineering, Xi’an Technological University, Xi’an 710021, China; yanglihong@xatu.edu.cn (L.Y.); yangzhiqiang@xatu.edu.cn (Z.Y.); zzgonglei@126.com (L.G.); wangwanjun126@126.com (W.W.); liyao_xatu@163.com (Y.L.); kinglywang3773@163.com (L.W.); chenzhili@xatu.cn (Z.C.); 2 95841Military Unit or Troop, Jiuquan 735000, China; 18584885046@wo.cn

**Keywords:** point cloud registration, 3D-SIFT feature extraction, fast point feature histogram, sampling consensus, symmetric target function, iterative closest point

## Abstract

In response to the issues of slow convergence and the tendency to fall into local optima in traditional iterative closest point (ICP) point cloud registration algorithms, this study presents a fast registration algorithm for laser point clouds based on 3D scale-invariant feature transform (3D-SIFT) feature extraction. First, feature points are preliminarily extracted using a normal vector threshold; then, more high-quality feature points are extracted using the 3D-SIFT algorithm, effectively reducing the number of point cloud registrations. Based on the extracted feature points, a coarse registration of the point cloud is performed using the fast point feature histogram (FPFH) descriptor combined with the sample consensus initial alignment (SAC-IA) algorithm, followed by fine registration using the point-to-plane ICP algorithm with a symmetric target function. The experimental results show that this algorithm significantly improved the registration efficiency. Compared with the traditional SAC−IA+ICP algorithm, the registration accuracy of this algorithm increased by 29.55% in experiments on a public dataset, and the registration time was reduced by 81.01%. In experiments on actual collected data, the registration accuracy increased by 41.72%, and the registration time was reduced by 67.65%. The algorithm presented in this paper maintains a high registration accuracy while greatly reducing the registration speed.

## 1. Introduction

Three-dimensional laser point cloud registration is widely used in various fields such as industrial production [1]; the medical and military fields, urban planning, cultural heritage protection, film, and entertainment [2]; and autonomous driving [3]. The latest research advancements across various fields are described in detail below. In the industrial production sector, registration techniques are utilized to detect workpiece defects and shapes [4], and terrestrial laser scanning methods have been applied in the digitalization of bridge infrastructure [5]. In the medical field, the application of point cloud registration in multimodal medical imaging has been explored, for example, in the integration of MRI and CT scan data [6], while in the field of cultural heritage preservation, the use of transformer-enhanced point cloud registration has been investigated for the protection of cultural sites such as the Terracotta Army [7]. Due to the influence of measurement devices, the measurement environment, and object occlusion, it is not feasible to acquire data on an object’s surface information in a single experiment. Thus, to obtain complete three-dimensional point cloud data of an object, it is usually necessary to measure it from multiple angles and then register or stitch the laser point clouds from different angles together to obtain a complete point cloud that contains the object’s information. Point cloud registration is the process of transforming point clouds from different angles through the calculation of rotation and translation matrices to the same coordinate system. Point cloud registration is a key challenge in computer vision and robotics, and is used to establish similarities between point cloud data captured from different viewpoints or at different instances in time and thus to generate accurate point cloud models.

The most classic method of point cloud registration is the iterative closest point (ICP) algorithm [8]. This algorithm iteratively finds the best transformation relationship between two-point clouds by minimizing the distance between corresponding points. However, this algorithm has problems such as sensitivity to initial transformation, slow convergence, poor robustness, and susceptibility to local optima. In recent years, researchers have proposed various optimization methods for the ICP algorithm. For example, the authors of [9] proposed a point-to-line ICP algorithm called PLICP, which uses the distance from points to lines to calculate the target function, considering the local structure information of the point cloud to improve registration accuracies. The authors of [10] proposed the generalized iterative closest point (GICP) algorithm, which introduces probability distributions and uses the covariance of the point cloud surface to construct the target function. By incorporating the normal vector information of the point cloud, it more effectively manages rotations and scale transformations. The authors of [11] proposed the outlier-trimmed iterative closest point (LieTrICP) algorithm, which is a robust registration method based on the outlier parameterization of two point sets, combining the advantages of outlier parameterization and trimmed ICPs and making the algorithm more robust and accurate. Compared to the ICP algorithm, the GICP and LieTrICP algorithms exhibit slower registration speeds but are relatively more complex to implement. The authors of [12] proposed the normal iterative closest point (NICP) algorithm, which considers the normal vector information of the point cloud and improves the registration process by combining the geometric features and local curvature information of the points. This method performs better in utilizing the local structure information of the point cloud, but it has stringent requirements for the initial pose. The authors of [13] proposed the global optimal iterative closest point (Go-ICP) algorithm, which integrates the local ICP algorithm into the branch and bound (BnB) algorithm, ensuring global optimality while accelerating the algorithm’s speed. The authors of [14] proposed an improved ICP algorithm, introducing a symmetric target function and considering the normals of corresponding points to improve the target function, further enhancing the accuracy and efficiency of the ICP algorithm. The authors of [15] proposed the vectorized generalized iterative closest point (VGICP) algorithm, utilizing the single instruction, multiple data (SIMD) instruction set of modern CPUs, converting traditional point-by-point calculations into vectorized calculations, designing a multicore parallel processing mechanism, and fully utilizing the computational power of multicore processors, thereby improving computational efficiency. However, it is essential to note that this method requires specific hardware support. The various optimization methods for ICP mentioned above have improved the registration effectiveness to a certain extent. However, they come with a higher computational complexity and require more sophisticated approaches to suppress noise, density variations, and outliers. This, in turn, will increase the computational burden. Other researchers have also proposed registration methods that are different from the ICP algorithm, such as the 3D normal distribution transform (3D-NDT) algorithm based on probability density distribution [16], which does not require the calculation of neighboring point pairs compared to the ICP algorithm and has faster registration speeds and higher accuracies when registering a large amount of data. Nevertheless, there is an inherent susceptibility to becoming trapped in local optima. The 4-point congruent sets (4PCS) algorithm [17], by constructing and matching the distance matrix of four-point pairs, reduces the spatial matching process, accelerating the registration process. This approach exhibits robustness against noise and outliers and is not sensitive to the initial pose. However, it is less effective for point clouds with high degrees of overlap compared to other algorithms.

Research into point cloud registration algorithms is progressing, with novel optimization techniques enhancing the efficacy of the registration process. Nonetheless, these advancements concurrently lead to increased computational complexity and necessitate the employment of more complex methodologies to mitigate the effects of noise, variations in point density, and the presence of outliers. Currently, researchers are further improving the registration accuracy and robustness by using coarse registration combined with fine registration. The authors of [18] proposed a feature descriptor based on the rotation volume ratio and then used coarse registration to obtain a good initial transformation matrix, finally completing the registration using an improved ICP algorithm. The authors of [19] proposed the fast point feature histogram (FPFH) descriptor and then used the improved k-dimensional (K-D) tree neighborhood query method (best-bin-first, BBF) for data dimensionality reduction, greatly reducing the running complexity. The authors of [20] proposed the sample consensus initial alignment (SAC-IA) algorithm for coarse registration, followed by ICP based on the point-to-plane distance. The aforementioned algorithm is feature-based [21] and does not directly compute point pairs between the source and target point clouds. Instead, it first extracts feature points and then describes them using feature descriptors. However, the selection of feature points and descriptors will definitely affect the registration outcome. Often, point clouds lack representativeness or have insufficient feature points, leading to a lower registration accuracy.

Based on the above literature review, this study combines the 3D scale-invariant feature transform (3D-SIFT) feature extraction algorithm and the FPFH feature descriptor to construct a fast registration algorithm for laser point clouds based on 3D-SIFT features. Unlike existing optimization-based methods, it makes two main contributions to improve the efficiency and accuracy of point cloud registration. (1) Innovation in feature extraction: By integrating normal vector preprocessing and the 3D-SIFT feature extraction algorithm, feature points with rich information are extracted. These feature points are more concise in quantity, avoiding the need for iteration over each point, effectively reducing the computational load and further decreasing the time and space complexity. (2) Optimization of the registration algorithm: A two-stage registration process is proposed, combining a sampling consistency-based (SAC-IA) coarse registration algorithm and a point-to-plane ICP (iterative closest point) fine registration algorithm based on a symmetric objective function. This improves the convergence speed and enhances the algorithm’s robustness to noise and outliers. This combination method not only leverages the scale invariance of 3D-SIFT in feature extraction and the ability of FPFH descriptors to capture local geometric features but also effectively addresses the limitations of traditional ICP algorithms through the coarse registration of SAC-IA algorithm and the fine registration of the ICP algorithm with a symmetric target function.

The rest of this paper is structured as follows. Section 2 introduces the proposed algorithm’s process. Section 3 provides a detailed explanation of the principles behind the five main steps of the proposed algorithm. Section 4 discusses the registration results and comparative results for the proposed algorithm with other algorithms on four sets of light detection and ranging (LiDAR) point cloud data. Finally, Section 5 focuses on the conclusions of this study.

## 2. Algorithm Design

**Figure 1:**
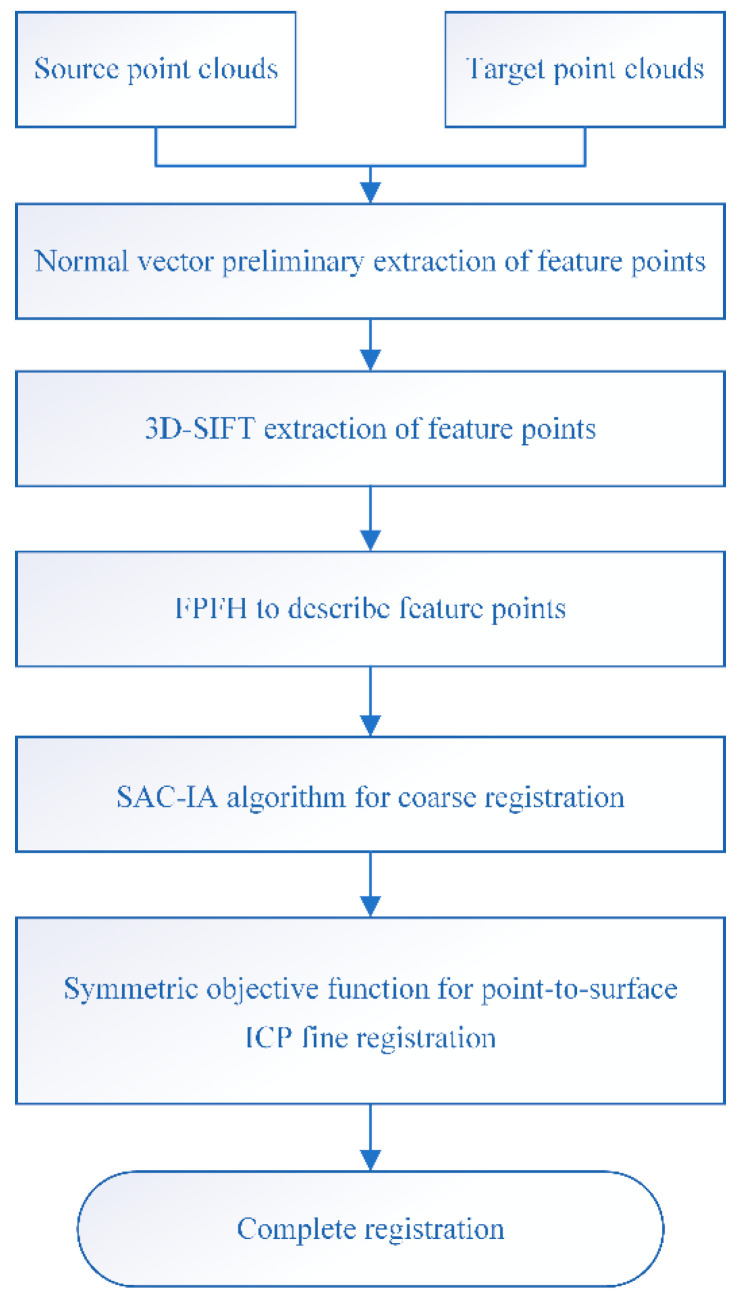
Algorithm flowchart.

The point cloud registration algorithm presented in this paper mainly includes five processes. Firstly, feature points are preliminarily extracted using the normal vector neighborhood angle, primarily by estimating the point cloud’s normal vectors with different neighborhood radii and extracting feature points based on the normal vector neighborhood angle. Thus, points that do not possess surface variations are removed. Secondly, the 3D-SIFT feature extraction algorithm is utilized to construct the scale-space of the point cloud after the initial feature point extraction. Detecting extremum points through the difference of Gaussian functions, extracting features at different scales, and precisely locating feature points through extremum detection and the Taylor fitting formula, further feature extraction is carried out to obtain points with rich, informative features. 3D-SIFT feature extraction provides key points and feature descriptors for subsequent steps, while also reducing the computational load for registration and enhancing computational efficiency. Next, the FPFH feature descriptor is used to describe the extracted feature points, capturing the local geometric features of point clouds, further enriching the information of the feature points, and making the matching between point clouds more accurate; then, the SAC-IA algorithm is used for coarse registration to obtain the optimal transformation matrix, which provides a favorable initial estimate for the iterative closest point (ICP) algorithm. This approach effectively reduces the computational load of the fine registration algorithm and enhances the success rate of registration. Finally, the point-to-plane ICP algorithm based on the symmetric target function is used, which provides constraints for point clouds with rich surface information, realizing the fine registration of the point cloud. The SAC-IA algorithm in the coarse registration phase and the symmetric target function ICP algorithm in the fine registration phase work together to accelerate the convergence of the registration process and improve the registration speed. By integrating process one (normal vector preprocessing) and process two (3D-SIFT) for feature extraction, high-quality feature points are obtained. The coarse and fine point cloud registration algorithms are combined through parts three, four, and five, achieving registration algorithm optimization. By combining the aforementioned algorithms, the computational load is reduced, robustness is improved, convergence is accelerated, and accuracy is enhanced, significantly increasing the efficiency and accuracy of point cloud registration. The flowchart of the algorithm is shown in Figure 1.

## 3. Algorithm Principle

### 3.1. Normal Vector Preliminary Feature Extraction

Due to the large volume of point cloud data, preliminary feature extraction using normal vectors can effectively reduce the number of points, eliminating those that do not exhibit surface variation, as well as some noisy points. A normal vector is a vector perpendicular to the object’s surface. First, the normal vectors of the point cloud are estimated using different neighborhood radii, and the principal component analysis method is used to estimate the surface normal vectors of the point cloud. Feature points are then extracted based on the normal vector neighborhood angle.

The normal estimation at any point $p_{i}$ in the point cloud data is approximated using the normal of a tangent plane relative to the estimated surface, that is, the least squares fitting plane. The direction vector of this plane is the normal vector at the corresponding point. The normal vector angle θ at any point $p_{i}$ in the point cloud is calculated using the least squares fitting plane method. Suppose there are k points in the neighborhood of each point, and the angle $\alpha_{ij}$ between the normal vectors of each point’s neighborhood is calculated as follows:

(1) $$\theta =\frac{1}{k}\sum_{j=1}^{k}\alpha_{ij}$$

where $\alpha_{ij}$ is the angle between the point cloud $p_{i}$ and the normal vectors of the neighboring points $p_{j}$. This average angle *θ* helps us to determine the surface variation at point $p_{i}$. If angle θ is greater than a predefined threshold, the point is considered to exhibit a surface variation and is extracted as a feature point.

The mathematical basis for this process lies in principal component analysis (PCA), which is a statistical procedure that uses an orthogonal transformation to convert a set of observations of possibly correlated variables into a set of values of linearly uncorrelated variables called principal components. The first principal component corresponds to the direction of the largest variance in the data, and each succeeding component corresponds to the direction of the second-largest variance, and so on.

**Figure 2:**
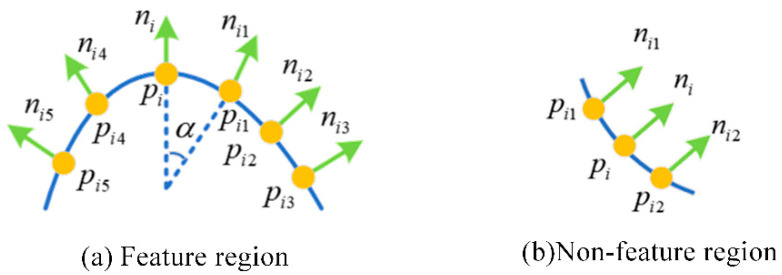
Feature region and nonfeature region normal vector angles.

By applying PCA to the neighborhood of each point in the point cloud, we can determine the direction of maximum variance, which represents the normal vector to the surface at that point. The angles between these normal vectors for neighboring points are then used to identify points with significant surface variations, which are extracted as feature points for further processing. As shown in Figure 2a, if the normal vector angles in a local area of the point cloud are large, this indicates that the area has significant undulations; otherwise, as shown in Figure 2b, if the normal vector angles do not change much, this indicates that the area is relatively flat. Selecting an appropriate normal vector angle threshold can determine the quality of feature extraction. Therefore, for different point cloud density distributions and point cloud noise conditions, suitable parameters can be chosen for preliminary extraction, thereby reducing the negative impact of this feature on the overall quality of point cloud registration.

### 3.2. Three-Dimensional-SIFT Feature Extraction

The 3D-SIFT algorithm is an extension of the SIFT algorithm into three-dimensional space. This algorithm has excellent performance in describing local features [22]. The 3D-SIFT feature extraction process includes the construction of scale-space, extrema detection, and extrema filtering. In 3D-SIFT, Gaussian functions are utilized to construct the scale-space of point clouds because of their desirable mathematical properties, such as localization and smoothness. The Gaussian convolution kernel is the only linear kernel capable of performing scale transformation, effectively simulating optical blur and the point spread function. Additionally, the responses at various scales can capture the features of the point cloud at different scales. In addition, we detected extrema, which are distinctive features within local regions, using the difference of Gaussian (DoG) function. In the process of extremum point filtering, the Taylor fitting formula is used to locate the extremum points accurately. Taylor fitting is a mathematical method that determines the precise location and scale of extremum points by locally approximating the function around the extremum points. This method can effectively reduce errors caused by noise or local variations, thereby enhancing the accuracy of keypoint detection. The main process is as follows:

(1) The scale-space of the point cloud is constructed. The scale-space of the source point cloud and the target point cloud is represented as follows:

(2) $$L_{p}(x,y,z,\sigma )=G_{p}(x,y,z,\sigma )*I_{p}(x,y,z,\sigma )$$

(3) $$L_{q}(x,y,z,\sigma )=G_{q}(x,y,z,\sigma )*I_{q}(x,y,z,\sigma )$$

Here, $G_{p}(x,y,z,\sigma )$ and $G_{q}(x,y,z,\sigma )$ are the variable-scale Gaussian functions of the source point cloud and the target point cloud, respectively. σ represents the scale-space factor, which determines the degree of Gaussian blur and the interval between different layers in the scale-space, is typically established based on the characteristics of the point cloud data and the desired scale of feature points. An appropriate factor can be determined through experimental or theoretical analysis to ensure the effective detection of key points at different scales, thereby capturing features that are invariant across scales; $I_{p}(x,y,z,\sigma )$ and $I_{q}(x,y,z,\sigma )$ are the curvature values of the source point cloud and the target point cloud.

(2) The extremum points in the scale-space are found; the difference of Gaussian (DoG) functions $G_{p}(x,y,z,\sigma )$ and $G_{q}(x,y,z,\sigma )$ are constructed for the source point cloud and the target point cloud.

(4) $$G_{p}(x,y,z,\sigma )=\frac{1}{2\pi \sigma^{2}}e^{-(x^{2}+y^{2}+z^{2})/(2\sigma^{2})}$$

(5) $$G_{q}(x,y,z,\sigma )=\frac{1}{2\pi \sigma^{2}}e^{-(x^{2}+y^{2}+z^{2})/(2\sigma^{2})}$$

By utilizing the DoG to detect feature points in each scale-space, the DoG function values of each sampling point are compared to those of its neighboring points. If the DoG function value of the sampling point is the maximum or minimum in the scale-space of the neighboring points, this sampling point is considered an extremum point in that scale-space.

(3) Screening of extremum points: The extremum points determined through the DoG function in both scale-spaces are taken as feature points. They are screened based on the Taylor fitting formula. The Taylor fitting formula for the DoG function in the scale-space is as follows:

(6) $$D(i)=D(i_{0})+\frac{\partial D{\left(i\right)}^{T}}{\partial i}i+\frac{1}{2}i^{T}\frac{\partial^{2}D(i)}{\partial i^{2}}i$$

In the point cloud space, i=(x,y,z,σ) represents any point, and $D(i_{0})$ represents the DoG function value at the extremum point $i_{0}$. By taking the derivative of Equation (6) and setting the first-order derivative to 0, the offset of the extremum point $\overset{\wedge }{I}$ is obtained as follows:

(7) $$\overset{\wedge }{I}=-\frac{\partial^{2}D^{-1}}{\partial I^{2}}\frac{\partial D}{\partial I}$$

After the offset is obtained from the above formula and added to the coordinates of the extremum point, a threshold is set for screening, and the stable extremum points are considered feature points. Since the effectiveness of the normal threshold value and 3D-SIFT largely depends on the appropriate parameter adjustment, it is necessary to set the threshold based on the specific point cloud density and noise level. The size of the threshold can be determined through multiple experiments or statistical methods.

Given the computational complexity of the 3D-SIFT feature extraction algorithm, performing preliminary feature extraction on the point cloud before applying this algorithm can alleviate this issue. Additionally, within the 3D-SIFT algorithm, a multiscale approach is adopted by constructing the scale-space of the point cloud at multiple levels; this further reduces the computational load, enabling improved applications in large-scale point clouds.

### 3.3. FPFH Feature Descriptor

Following the feature extraction algorithm mentioned above, the FPFH feature descriptor is used to describe the feature points. It is constructed based on the normal vectors and curvature information within the neighborhood of a point cloud, capturing the local geometric features of the point cloud. The FPFH descriptor is not solely dependent on the surface curvature of the point cloud, it also significantly relies on normal vector information. Calculation of the FPFH feature descriptor encompasses the calculation of normal vectors and curvatures and relative orientations and curvatures as well as the construction of feature histograms.

**Figure 3:**
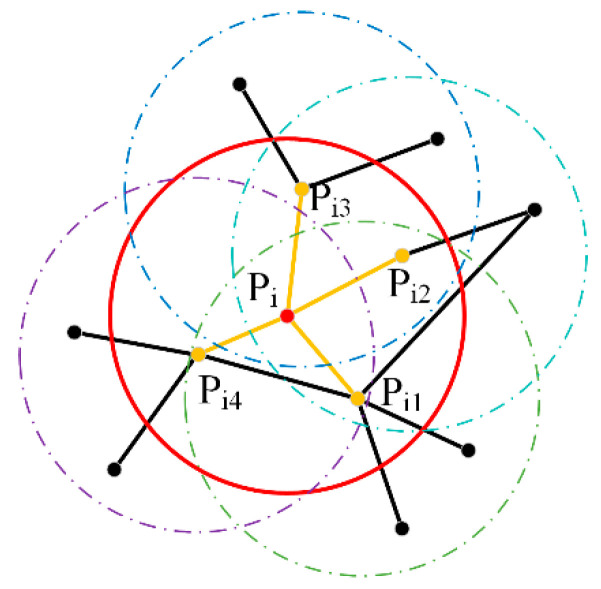
Principle of FPFH calculations.

To construct the FPFH descriptor, we first calculate the normal vector and curvature for each point within a specified radius k. For a given point $p_{i}$, let $n_{i}$ be its normal vector and k be its curvature. The normal vector $n_{i}$ is estimated using principal component analysis (PCA) on the points within the neighborhood of $p_{i}$. The curvature k is calculated based on the distribution of the neighboring points relative to $n_{i}$. Next, we compute the relative orientation and curvature between point $p_{i}$ and all its neighboring points $p_{j}$ within the neighborhood. The relative orientation is determined by the angle between $n_{i}$ and $n_{j}$, and the curvature is determined by the distance from $p_{j}$ to the plane defined by $p_{i}$ and $n_{i}$. These parameters are inserted into a 33-dimensional feature histogram to determine the correspondence between feature points. The working principle of the FPFH descriptor for point cloud feature extraction is shown in Figure 3. The red points are the query center points, the yellow points are the neighboring points within their radii, and the black points are the remaining points in the space. The angles between the center points and the neighboring points can be calculated through the yellow lines, and the FPFH values can be further computed.

The steps for calculating the FPFH feature descriptor are as follows:

(1) The relative relationship between any point $P_{i}$ and its neighboring points is calculated within a certain radius k, and the simple point feature histogram is constructed, denoted as SPFH ($P_{i}$).
(2) Based on the weight ratio of each point within the neighborhood, the FPFH feature value of point $P_{i}$ is recalculated according to the following formula:
  (8) $$\mathrm{FPFH}(P_{i})=\mathrm{SPFH}(P_{i})+\frac{1}{k}\sum_{j=1}^{k}\frac{1}{w}\mathrm{SPFH}(P_{j})$$

Here, $\mathrm{FPFH}(P_{i})$ is the FPFH descriptor, and w is the distance between the query point $P_{i}$ and its neighboring point $P_{k}$, which is used to evaluate the relationship between the point pair ($P_{i}$,$P_{k}$). The initial task of the FPFH descriptor is to determine a neighborhood for each feature point, which is typically a sphere of radius R that includes the feature point and surrounding points (*k* nearest neighbors). The size of the neighborhood determines the locality of the descriptor, so by choosing an appropriate number of neighboring points, the accuracy and efficiency of registration can be enhanced.

### 3.4. SAC-IA Coarse Registration Algorithm

The calculated FPFH is used with the SAC-IA algorithm to find corresponding points between the source point cloud and the target point cloud. The steps of the SAC-IA registration process are as follows:

(1) n sample data points are randomly selected from the source point cloud P, and the distance between any two points must be greater than the set distance threshold $w_{d}$ to ensure that the sample data points have different FPFH features.
(2) Using the FPFH feature as a constraint, the target point cloud Q is searched for in the point cloud data with similar FPFH features to the source point cloud P, where similar points form corresponding points, and a set of corresponding points is randomly formed.
(3) Based on the corresponding point pairs ($p_{i}$,$q_{i}$) from the two point clouds, the translation and rotation matrices are calculated, and the distance error and the Huber penalty function formula are used to determine whether it is the optimal transformation matrix:
  (9) $$H(l_{i})=\left\{\begin{array}{ccc} \frac{1}{2}l_{i}^{2} & ; & ∥l_{i}∥<w_{d}\\ \frac{1}{2}m_{l}(∥l_{i}∥-w_{d}) & ; & ∥l_{i}∥>w_{d} \end{array}\right.$$

Among them, $l_{i}$ represents the distance difference between the sample’s corresponding data points $p_{i}$ and $q_{i}$ before and after transformation; $w_{d}$ is the set distance threshold.

The above process is repeated and iterated until the convergence condition is met or the maximum number of iterations is reached, which is considered the optimal transformation.

By setting a distance threshold between point pairs, it is possible to effectively eliminate outliers or rogue points effectively. Setting a maximum number of iterations helps to find the global optimum, avoiding being trapped in local optima.

### 3.5. Symmetric Target Function ICP Fine Registration

**Figure 4:**
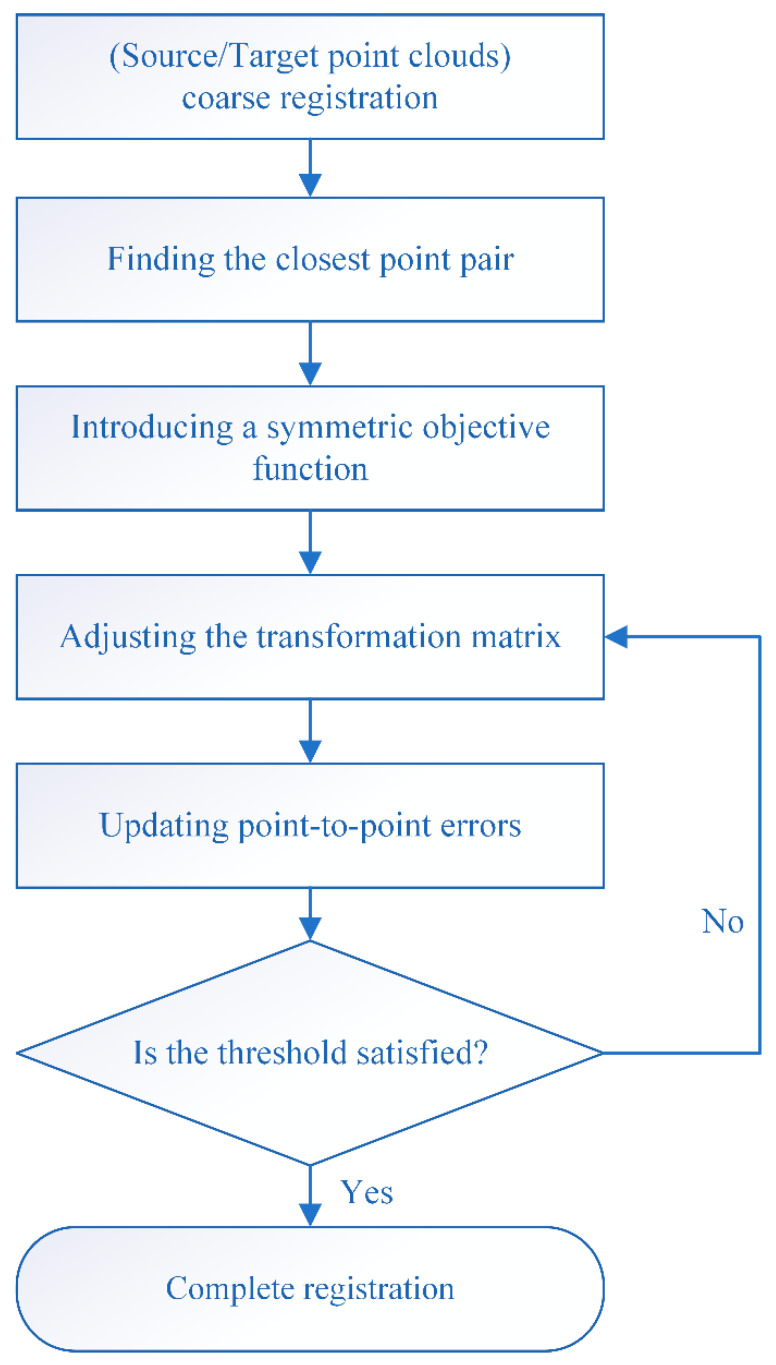
Symmetric target function ICP algorithm flowchart.

Due to the slow convergence and susceptibility to local optima in traditional ICP algorithms, the ICP algorithm iteratively obtains the optimal transformation matrix using least squares and uses a point-to-point metric for registration. However, the ideal state of the algorithm is that both the source point cloud and the target point cloud have corresponding points. If there are any missing data in the point cloud, this will affect the accuracy and convergence of the algorithm. The local geometric structure of the surface is ignored, and the target function cannot obtain the precise overlap degree of a single point. Therefore, this algorithm adopts an improved symmetric target function point-to-plane ICP algorithm, which introduces a symmetric target function and combines the surface information of the point cloud, providing constraints for point clouds with rich surface information. The flowchart of the fine registration algorithm is shown in Figure 4.

Using the point cloud after coarse registration, the nearest point pairs ($p_{i}$,$q_{i}$) between the source point cloud and target point cloud are found, and then the symmetric target function is used to calculate the error of the point pair ($p_{i}$,$q_{i}$). The specific principle of the symmetric target function is as follows.

The symmetric target function is symmetric, meaning that if the two point clouds are the result of scanning the same object, each point $P_{i}$ in the point cloud exists with a corresponding point $Q_{i}$ in the point cloud, and vice versa. The symmetric target function E is defined as minimizing the measure of distances between all point pairs ($p_{i}$,$q_{i}$), with the formula as follows:

(10) $$E=\sum_{i}{\left\|(p_{i}-q_{i})\cdot \left.\left(n_{p}+n_{q}\right)\right\|\right.}^{2}$$

In the formula, $n_{p}$ and $n_{q}$ are the normal vectors of points $p_{i}$ and $q_{i}$, and $\left\|\left.\cdot \right\|\right.$ represents their Euclidean distance.

**Figure 5:**
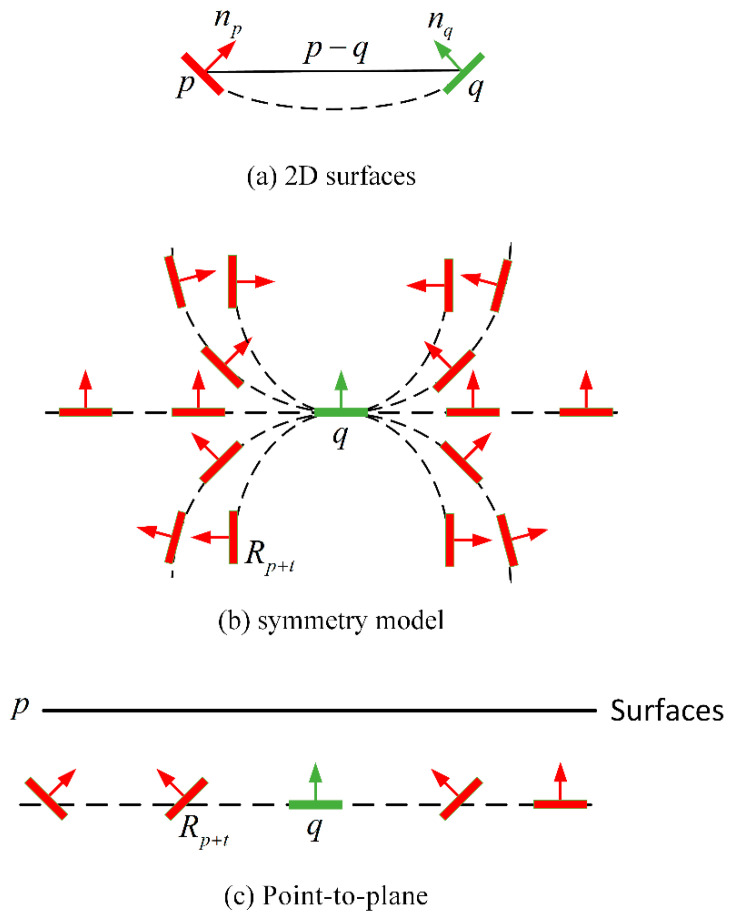
Symmetric target function schematic diagram.

The geometric meaning of this symmetric target function is shown in Figure 5.

The vector between any arbitrary sampling points p and q on the arc is perpendicular to the sum of the normal $n_{p}+n_{q}$, and the symmetric function value is 0, as shown in Figure 5a. Figure 5b illustrates how a transformation is applied to sampling points p (the red parts) and q on a 2D surface, where the transformed points are geometrically consistent with the original point pairs, and they can be symmetric about some axis or center, where $R_{p+t}$ represents the point after the transformation of the point cloud. The transformation of point p (the red part) is achieved by considering its normal $n_{p}$, and in the point-to-plane distance metric, the target function calculates the distance from point p to the plane that passes through point q with a normal $n_{q}$, as shown in Figure 5c. The symmetric target function provides a geometric constraint that not only requires the distance between points to be minimized but also requires consistent normal directions, which helps capture the local geometric features of the point cloud, thereby improving the accuracy of the registration.

Finally, numerical optimization methods (such as the Newton method) are used to adjust the transformation matrix to minimize the symmetric target function. Based on the results of the optimization algorithm, the best rigid body transformation is found after multiple iterations. The process then checks whether the algorithm meets the set convergence criteria or reaches the maximum number of iterations to complete the fine registration process of the point cloud. The function takes into account the surface information of the point clouds, necessitating that corresponding points in the source and target point clouds not only have the minimum distance but also have consistent normal vector directions.

## 4. Experimental Results and Analysis

### 4.1. Experimental Data

To verify the effectiveness and practicality of the algorithm presented in this paper, verification experiments were conducted using both public datasets and actual collected data. The actual collected dataset was constructed using point cloud data obtained from LS128S2 series 1550 nm hybrid solid-state LiDAR equipment provided by Shenzhen Leishen Intelligent Equipment Co., Ltd., Shenzhen, China. Four samples were selected as the dataset, namely, an indoor table (Sample 1), an outdoor car 1 (Sample 2), an outdoor car 2 (Sample 3), and an outdoor stele (Sample 4). The number of points in the point clouds are as follows: Sample 1 (8064, 8193), Sample 2 (11,985, 13,478), Sample 3 (13,003, 8409), and Sample 4 (7507, 5543). The selected public dataset consists of four sets of data from the 3D-Match dataset: “living room 1” (47,553, 53,064), “living room 2” (127,241, 159,067), “office 1” (135,151, 95,443), and “office 2” (406,192, 365,570). This algorithm was implemented using a Windows 11 operating system, the development environment of Microsoft Visual Studio 2022, and PCL 1.13 (a third-party point cloud library) using the C++ language.

### 4.2. Evaluation Metrics

To verify the registration effect of the algorithm, the root mean square error (RMSE) of the point-to-point distance in the overlapping part and the algorithm’s running time (time in seconds) are used as the evaluation metrics for the accuracy of point cloud registration. The execution time of an algorithm directly reflects its computational efficiency and is a key factor in evaluating the efficiency of point cloud registration. The RMSE is a measure of “mean error” that evaluates the degree of variability of the data and is commonly used in point cloud registration to assess the performance of registration algorithms by comparing the differences between the source point cloud and the target point cloud to measure registration accuracies. The RMSE refers to the average of the sum of squares of the distances between the corresponding points of two point clouds. The smaller the RMSE, the better the registration effect. The formula for the RMSE is as follows:

(11) $$\mathrm{RMSE}=\sqrt{\frac{1}{m}{\sum_{i=1}^{m}\left\|p_{i}\right.-\left.q_{i}\right\|}^{2}}$$

In this formula, m is the number of corresponding points between point cloud P and point cloud Q; $p_{i}$ and $q_{i}$ are the corresponding points of the two point clouds; $\left\|p_{i}\right.-\left.q_{i}\right\|$ is the Euclidean distance between $p_{i}$ and $q_{i}$.

### 4.3. Feature Point Extraction Results

This algorithm combines preliminary feature extraction using normal vectors with 3D-SIFT feature extraction to extract features from the point cloud. High-quality feature points are extracted, which not only achieves high registration accuracies but also reduces the running time of the registration algorithm.

**Figure 6:**
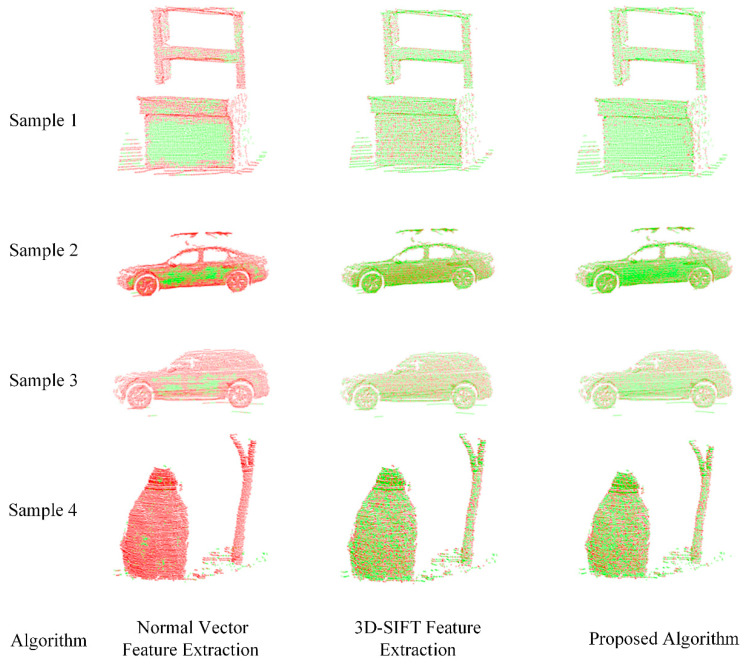
Comparison of the visualized results of feature point extraction.

To verify the effect of feature point extraction, the normal vector feature extraction algorithm, the 3D-SIFT feature extraction algorithm, and the feature extraction algorithm presented in this paper were compared. Feature points were extracted from the four sets of real collected data, with the number of extracted feature points, as shown in Table 1. The comparison effect is shown in Figure 6, where the green point cloud represents the original point cloud, and the red point cloud represents the extracted feature points.

From the above results, it can be observed that the normal vector feature extraction algorithm extracts too many feature points that are also densely distributed. Since normal vector feature extraction can effectively capture the geometric features of objects, such as the central parts in samples 1, 2, and 3, and there are fewer geometric features in sample 4, fewer feature points are filtered out. This algorithm is sensitive to noise and the quality of the data; if the normal vector angle is large, it is not conducive to extracting effective point cloud features.

The 3D-SIFT feature extraction algorithm extracts fewer feature points than the normal vector feature extraction algorithm, but it captures more geometric feature points that are relatively dense. Using the feature extraction algorithm presented in this paper, not only is the density of the point cloud reduced and a sparser set of point cloud features is extracted, but it also retains the surface characteristics of the original point cloud, resulting in the extraction of high-quality feature points.

### 4.4. Analysis of Point Cloud Registration Using the Actual Collected Dataset

To verify the effectiveness and accuracy of the improved point cloud registration algorithm, four different algorithms were designed for comparison with the algorithm presented in this paper under the same conditions. The four algorithms are the traditional SAC−IA+NDT algorithm, the SAC−IA+ICP algorithm, SAC−IA+SICP (SAC-IA+ point-to-plane ICP algorithm with a symmetric target function), the 3DSC (3D Semantic Consensus)-RANSAC (Random Sample Consensus)+ICP algorithm, and the proposed algorithm. During algorithm design, the selection and optimization of parameters are crucial steps to maintain the performance of the algorithm. A variety of parameters require adjustment and optimization to accommodate different datasets or diverse scenarios. To ensure the comparability of the algorithms, the common parameters in the four algorithms were set to be consistent, with specific parameter settings as follows: a neighborhood radius of 0.05, ICP iteration number of 50, convergence threshold of 0.01, the maximum distance between corresponding points of 0.1, random sample consensus inlier threshold of 0.1, and normal vector angle threshold of 5°. This optimization is primarily based on the density and distribution of the point cloud, assessing the performance of different parameter settings. Through numerous experimental adjustments, the optimal combination of parameters was identified.

**Figure 7:**
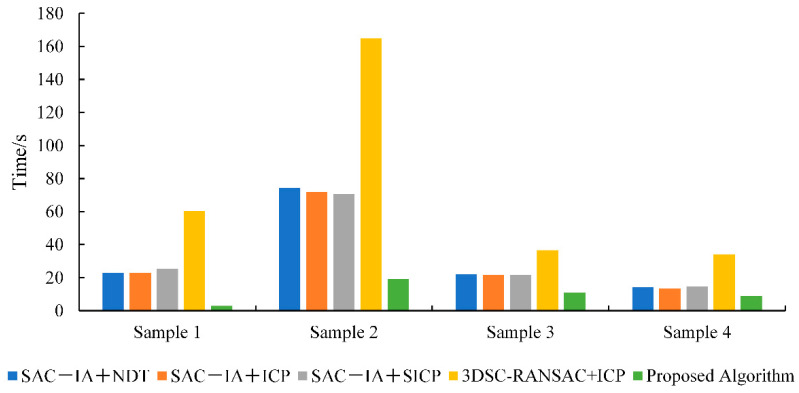
Comparison chart of the registration running times of each algorithm.

Table 2 provides the registration running times for the four samples of each algorithm. Compared to the SAC−IA+NDT algorithm, the registration time increased by 68.64%; compared to the SAC−IA+ICP algorithm, it increased by 67.65%; compared to the SAC−IA+SICP algorithm, it increased by 68.30%; and compared to the 3DSC−RANSAC+ICP algorithm, it increased by 86.49%. Figure 7 shows a comparison of the registration running times of each algorithm. It can be observed that the proposed algorithm has the shortest registration time, achieving the effect of fast point cloud registration.

**Figure 8:**
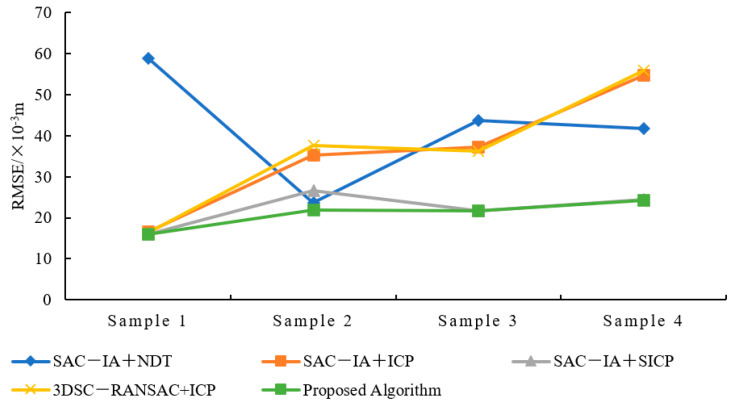
Accuracy comparison chart for various algorithms.

The results from Table 3 on the registration accuracy of various algorithms and Figure 8 on the accuracy comparison chart show that the algorithm presented in this paper has the best registration accuracy. Compared with the SAC−IA+NDT algorithm, the proposed algorithm’s registration accuracy increased by 50.07%. Compared with the SAC−IA+ICP algorithm, the registration accuracy increased by 41.72%. Compared with the SAC−IA+SICP algorithm (SAC-IA+ point-to-plane ICP algorithm with a symmetric target function), the registration accuracy increased by 5.51%. Compared with the 3DSC−RANSAC+ICP algorithm, the registration accuracy increased by 42.59%.

The registration accuracy for Sample 1 is significantly improved by the algorithm presented in this paper, while the improvements are not as pronounced for Samples 3 and 4. This discrepancy is attributed to the fact that Sample 1 possesses more distinct geometric features and a more regular distribution. The point cloud data of samples 3 and 4 exhibit more complex geometric structures, and Sample 4 contains a larger number of outliers, making it difficult for the algorithm to identify and utilize key features for registration effectively. In contrast, the point cloud in Sample 2 exhibits a moderate distribution of geometric feature information and outlier points.

**Figure 9:**
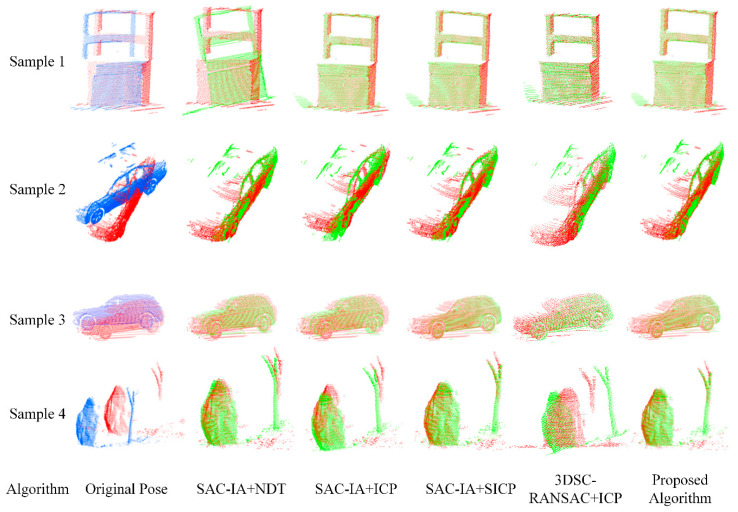
Comparison of point-cloud registration visualization results for the actual collected dataset.

The visualization effects of the registration results of each algorithm are shown in Figure 9, where the source point cloud is blue, the target point cloud is green, and the registered point cloud is also green. From the results, it can be observed that for point clouds with large initial position differences, the SAC−IA+NDT algorithm has a relatively large overall accuracy error, with only Sample 2 successfully registered and all others failing. The SAC−IA+ICP algorithm also has a relatively large accuracy error, with only Sample 1 successfully registered. The accuracy of the 3DSC−RANSAC+ICP algorithm is similar to that of the SAC−IA+ICP algorithm. The SAC-IA+ (point-to-plane with a symmetric target function) ICP algorithm has a relatively lower registration accuracy error, with all samples successfully registered, and the difference compared to the proposed algorithm is relatively small, but the proposed algorithm significantly reduces the registration time and improves registration efficiencies. Therefore, the registration effect of this algorithm is significantly improved compared to the other algorithms.

### 4.5. Analysis of Point Cloud Registration of Public Datasets

In this experiment, four sets of data from the 3D-Match dataset were utilized for registration validation. Given the large number of point clouds in the dataset, a voxel downsampling method was first applied for preprocessing to reduce the point cloud data while preserving complete features, thereby further enhancing the registration speed. The choice of voxel size directly affects the precision of point cloud data and the processing efficiency. A smaller voxel size can retain more details but increase the computational load; a larger voxel size reduces the computational load but may sacrifice some detailed information. Voxel size plays a crucial role in the preprocessing step, as it not only affects the speed and efficiency of data processing but also directly influences the precision of subsequent analyses and the quality of results. Choosing the appropriate voxel size involves a trade-off between the specific application requirements and the computational resources. The voxel size used in this experiment was 0.01, and the registration time and accuracy of five algorithms were compared at the level.

**Figure 10:**
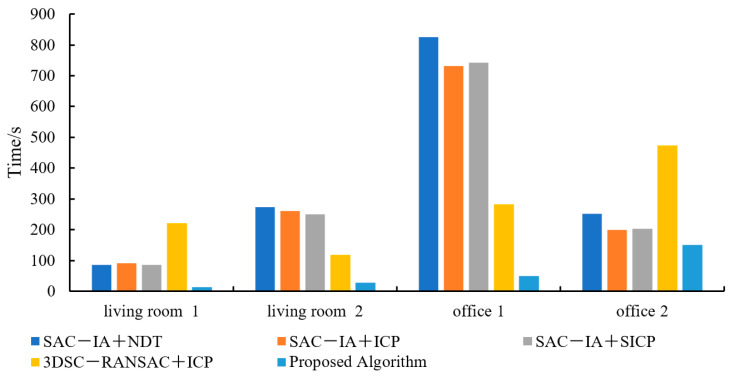
Comparison of the registration running times of each algorithm.

Table 4 presents the registration running times of the algorithms and Figure 10 shows a comparison. Compared to the SAC−IA+NDT algorithm, the registration time increased by 83.05%; compared to the SAC−IA+ICP algorithm, it increased by 81.01%; compared to the SAC−IA+SICP algorithm, it increased by 81.03%; and compared to the 3DSC−RANSAC+ICP algorithm, it increased by 77.81%. Due to the high number of point clouds in the public dataset, the algorithm in this study extracted good feature points through multiple rounds of feature extraction, reducing the point cloud input for the registration algorithm and thus significantly improving the registration time.

**Figure 11:**
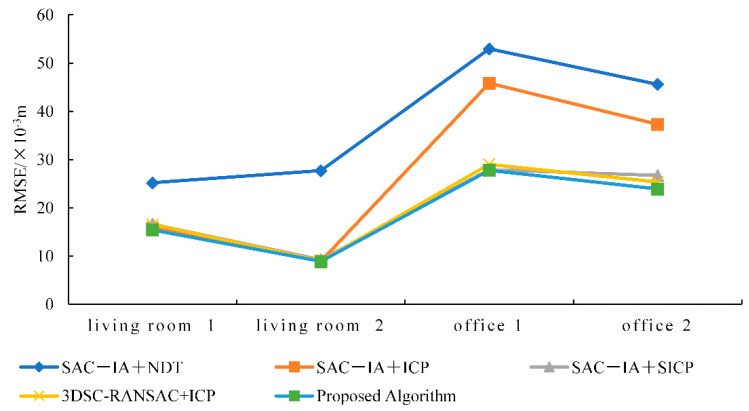
Accuracy comparison for various algorithms.

The results shown in Table 5 and Figure 11 regarding the registration accuracy of various algorithms indicate that the algorithm presented in this paper has improved the registration accuracy by 49.50%, 29.55%, 5.576%, and 4.85% compared to the SAC−IA+NDT algorithm, the SAC−IA+ICP algorithm, the SAC−IA+SICP algorithm, and the 3DSC−RANSAC+ICP algorithm, respectively. The SAC−IA+NDT algorithm can achieve registration without the need to rematch corresponding points at each iteration; thus, it is fast and accurate when dealing with massive data. However, when registering medium- and small-sized objects, the registration effect is not very good; hence, the algorithm in this paper presents a significant improvement with regard to registration accuracy. The SAC−IA+ICP algorithm has a variable registration accuracy, which is due to the lack of feature points in the office 1 and office 2 data, and this is also affected by other factors. The other two algorithms exhibit no significant improvements in accuracy, as they are inherently more suitable for the registration of medium- and small-sized objects. Hence, the improvement in accuracy in this paper is not significant.

**Figure 12:**
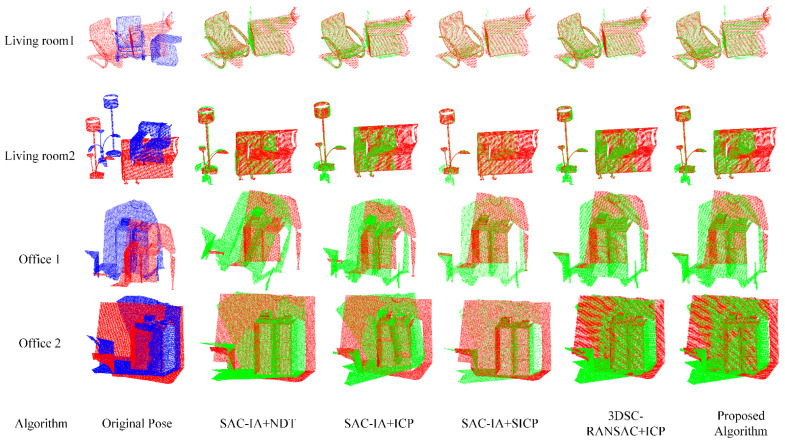
Comparison of point cloud registration visualizations for public datasets.

A visualization of the registration results for this dataset is shown in Figure 12. It is evident from the figure that the SAC−IA+NDT algorithm has a high registration accuracy error, with only half of the registrations being successful. This is due to the inherently lower precision of the algorithm itself. In the SAC−IA+ICP algorithm, the accuracy error is also relatively large. The other algorithms all successfully achieved registration. In addition to ensuring registration accuracy, the algorithm presented in this paper significantly reduced the time required, especially for datasets with a large number of point clouds, where the effect is more pronounced. The experimental results indicate that the algorithm presented in this paper has significant advantages in improving the efficiency and accuracy of point cloud registration. However, the performance of the algorithm is influenced by parameter settings. Future efforts will be dedicated to further optimizing the mechanisms of parameter selection and threshold setting to reduce the reliance on experience.

## 5. Conclusions

To address the issues of slow convergence and susceptibility to local optima in traditional point cloud registration algorithms, this paper introduces a fast registration algorithm for laser point clouds based on 3D-SIFT features. Initially, feature points are preliminarily extracted using a normal vector threshold, followed by the further extraction of high-quality feature points using the 3D-SIFT algorithm, effectively reducing the number of registrations needed and improving the efficiency of point cloud registration. Subsequently, a coarse registration of the point cloud is performed using the FPFH descriptor in conjunction with the SAC-IA algorithm, and finally, a fine registration is carried out using the point-to-plane ICP algorithm with a symmetric target function. Comparative analyses with the SAC−IA+NDT algorithm, SAC−IA+ICP algorithm, 3DSC−RANSAC+ICP algorithm, and SAC-IA+ (point-to-plane with a symmetric target function) ICP algorithm demonstrated that this algorithm has significantly improved registration efficiencies. Compared to the SAC−IA+ICP algorithm, the registration accuracy of the proposed algorithm increased by 29.55% on public datasets, and the registration time was reduced by 81.01%. In the experiments on real collected data, the registration accuracy increased by 41.72%, and the registration time was reduced by 67.65%. The number of point clouds in the public dataset is significantly higher than that in the actual collected dataset; hence, the improvement in time efficiency is more pronounced. The proposed algorithm not only maintains a high registration accuracy but also significantly speeds up the registration process.

This paper mainly describes the accuracy of point cloud registration through the global indicator of RMSE. If it is necessary to reflect the accuracy of local area registration results, some local feature matching measurement methods can be introduced, such as calculating the key point matching rate, feature edge alignment degree, and local root mean square error within local areas, so as to more finely reflect the registration effect of local areas.

The algorithm in this paper relies on environmental selection in terms of parameter selection and threshold setting and may have limitations when dealing with complex data sets. It is often difficult to find the optimal parameter combination when facing data of different scales, different noise levels, and different structural features. The aforementioned issues will be addressed in future research by further optimizing the mechanisms for parameter selection and threshold setting. For example, adaptive technologies relying on algorithms such as particle swarm optimization can dynamically adjust algorithm parameters according to the characteristics of input data, thereby better coping with the diversity of different scenarios and data. In the future, consideration will be given to combining with deep learning. Deep learning models, such as convolutional neural networks (CNN) and recurrent neural networks (RNN), can learn discriminative feature representations from large amounts of data. These features better reflect the essential structure and inherent laws of the data, thereby enabling the automatic extraction of complex feature points and providing stronger support for subsequent algorithm processing. At the same time, the features and patterns learned by deep learning models from data can also guide the parameter adjustment strategies in adaptive technologies. By analyzing the performance of deep learning models on different data and extracting key feature indicators, such as the stability and discriminability of features, parameters of adaptive algorithms can then be dynamically adjusted according to these indicators, enabling better collaborative work between the two, thereby further enhancing the accuracy and efficiency of point cloud registration. We look forward to achieving a more efficient, accurate, and robust point cloud registration algorithm in future studies.

## Figures and Tables

**Table 1:** Number of feature points extracted using each algorithm.

Model	Original Point Clouds	Normal Vector Feature Extraction	3D-SIFT Feature Extraction	Proposed Algorithm
Sample 1	8064	4469	3272	1958
Sample 2	20,091	14,986	10,412	7950
Sample 3	10,567	8662	6559	5642
Sample 4	7507	7101	4928	4685

**Table 2:** Registration running time for four models of each algorithm (in seconds).

Model	SAC−IA+NDT	SAC−IA+ICP	SAC−IA+SICP	3DSC−RANSAC+ICP	Proposed Algorithm
Sample 1	22.900	22.831	25.442	60.246	2.921
Sample 2	74.420	71.783	70.618	165.006	19.164
Sample 3	22.118	21.625	21.611	36.703	10.996
Sample 4	14.275	13.356	14.579	34.202	8.839

**Table 3:** Registration accuracy of the five algorithms: RMSE/m.

Model	SAC−IA+NDT	SAC−IA+ICP	SAC−IA+SICP	3DSC−RANSAC+ICP	Proposed Algorithm
Sample 1	58.7613 × 10^−3^	16.5948 × 10^−3^	16.0429 × 10^−3^	16.3027 × 10^−3^	16.0186 × 10^−3^
Sample 2	23.5844 × 10^−3^	35.2946 × 10^−3^	26.5466 × 10^−3^	37.5196 × 10^−3^	21.7988 × 10^−3^
Sample 3	43.7590 × 10^−3^	37.2298 × 10^−3^	21.6974 × 10^−3^	36.2834× 10^−3^	21.6182 × 10^−3^
Sample 4	41.7201 × 10^−3^	54.6610 × 10^−3^	24.3818 × 10^−3^	55.8458× 10^−3^	24.3426 × 10^−3^

**Table 4:** Registration running time for four models of each algorithm (in seconds).

Model	SAC−IA+NDT	SAC−IA+ICP	SAC−IA+SICP	3DSC−RANSAC+ICP	Proposed Algorithm
living room 1	85.689	91.645	85.251	220.538	13.752
living room 2	274.386	260.334	251.066	118.648	28.281
office 1	824.458	730.709	743.023	283.169	50.505
office 2	251.888	198.876	203.581	474.318	150.812

**Table 5:** Registration accuracy of the five algorithms: RMSE/m.

Model	SAC−IA+NDT	SAC−IA+ICP	SAC−IA+SICP	3DSC−RANSAC+ICP	Proposed Algorithm
living room 1	25.2053 × 10^−3^	15.7957 × 10^−3^	16.6153 × 10^−3^	16.5455 × 10^−3^	15.4274 × 10^−3^
living room 2	27.7137 × 10^−3^	8.9593 × 10^−3^	9.2613 × 10^−3^	9.0130 × 10^−3^	8.8923 × 10^−3^
office 1	52.9339 × 10^−3^	45.8435 × 10^−3^	27.9142 × 10^−3^	29.0209× 10^−3^	27.7879 × 10^−3^
office 2	45.5891 × 10^−3^	37.3221 × 10^−3^	26.7287 × 10^−3^	25.3336× 10^−3^	23.9222 × 10^−3^

## Data Availability

The data used to support the findings of this study are available from the corresponding author upon request.

## References

[1] Xue S., Lv N., Shen Y., Liu Z., Guo J. (2019). Identification method for machine workpiece based on laser 3D point cloud. Infrared Laser Eng..

[2] Xiong S., Wang Q., Liu G. (2022). A Comprehensive Review on the Development and Current Status of 3D Reconstruction Technology. Comput. Knowl. Technol..

[3] Li G., Shao S., Xun L., Xiang L. (2021). 3D multi-object tracking algorithm based on laser point cloud coordinate system. Laser Infrared.

[4] Wang S., Deng Z., Ge J., Wei L. (2023). Research Progress on Point Cloud Registration Technology for Industrial Three-Dimensional Inspection. Diam. Abras. Eng..

[5] Mohammadi M., Rashidi M., Mousavi V., Yu Y., Samali B. (2022). Application of TLS Method in Digitization of Bridge Infrastructures: A Path to BrIM Development. Remote Sens..

[6] Li W., Zeng Q., Li Y. (2019). Real-time Registration Simulation of Multimodal Medical Image Outer Boundary Point Cloud Data. Comput. Simul..

[7] Wang Y., Zhou P., Geng G., An L., Zhou M. (2024). Enhancing point cloud registration with transformer: Cultural heritage protection of the Terracotta Warriors. Herit. Sci..

[8] Besl P.J., McKay N.D. (1992). Method for Registration of 3-D Shapes.

[9] Censi A. An ICP variant using a point-to-line metric. Proceedings of the 2008 IEEE International Conference on Robotics and Automation.

[10] Segal A.V., Hähnel D., Thrun S. (2009). “Generalized-ICP.” Robotics: Science and Systems.

[11] Dong J., Peng Y., Ying S., Hu Z. (2014). LieTrICP: An improvement of trimmed iterative closest point algorithm. Neurocomputing.

[12] Serafin J., Grisetti G. NICP: Dense normal based point cloud registration. Proceedings of the IEEE International Conference on Intelligent Robots and Systems (IROS).

[13] Yang J., Li H., Campbell D., Jia Y. (2016). Go-ICP: A globally optimal solution to 3D ICP point set registration. IEEE Trans. Pattern Anal. Mach. Intell..

[14] Rusinkiewicz S. (2019). A symmetric objective function for ICP. ACM Trans. Graph. (TOG).

[15] Koide K., Yokozuka M., Oishi S., Banno A. (2020). Voxelized gicp for fast and accurate 3D point cloud registration. EasyChair Prepr..

[16] Magnusson M., Lilienthal A., Duckett T. (2007). Scan registration for autonomous mining vehicles using 3D-NDT. J. Field Robot..

[17] Ge X. (2017). Automatic markerless registration of point clouds with semantic-keypoint-based 4-points congruent sets. ISPRS J. Photogramm. Remote Sens..

[18] Xiong F., Dong B., Huo W., Min P., Liqun K., Xie H. (2020). A Local Feature Descriptor Based on Rotational Volume for Pairwise Registration of Point Clouds. IEEE Access.

[19] Wu P., Li W., Yan M. (2020). 3D Scene Reconstruction based on improved ICP algorithm. Microprocess. Microsyst..

[20] Shen X., Ge Z., Gao Q., Sun H., Tang X., Cai Q. A point cloud registration algorithm for the fusion of virtual and real maintainability test prototypes. Proceedings of the 2022 3rd International Conference on Computing, Networks and Internet of Things (CNIOT).

[21] Wu A., Ding Y., Mao J., Zhang X. (2022). A Fast Point Clouds Registration Algorithm Based on ISS-USC Feature for the 3D Laser Scanner. Algorithms.

[22] Bo Q., Xiyu S. (2024). ICP Registration Algorithm Based on SIFT Feature Point Extraction. J. Shenyang Ligong Univ..

